# Colloidal Quantum Dots for Nanophotonic Devices

**DOI:** 10.3390/ma17112471

**Published:** 2024-05-21

**Authors:** Menglu Chen, Qun Hao

**Affiliations:** 1 School of Optics and Photonics, Beijing Institute of Technology, Beijing 100081, China; menglu@bit.edu.cn; 2 Physics Department, Changchun University of Science and Technology, Changchun 130022, China

Colloidal quantum dots (CQDs) have unique advantages in the wide tunability of visible-to-infrared emission wavelength and low-cost solution processibility [1,2,3,4,5]. Therefore, they have become an important class of materials with great potential for applications in fields such as biological medicine [6,7], optoelectronics [8,9,10,11,12], and quantum information [13]. The performance of CQD-based photovoltaic and light-emitting devices has become competitive with other state-of-the-art materials [14,15,16]. Narrow-band semiconductor CQDs also hold unique promise for infrared technologies [17,18]. Thus, new and in-depth insights into CQD growth, chemical transformations, and physical properties would benefit not only the purely fundamental side but also commercialization. This Special Issue (SI), “Colloidal Quantum Dots for Nanophotonic Devices”, presents recent and CQD-related information ranging from CQD material chemistry and characterization to processing and device fabrication. This SI contains ten articles, including seven research articles and three review articles. This editorial aims to summarize the publications included in this SI.

CQDs have the advantages of a broad spectral tuning range, low preparation cost, and compatibility with silicon-based readout integrated circuits via solution processing [1,5,8,19,20,21]. As a result, CQD photodetectors have become a research hotspot in recent years. Lead chalcogenide CQDs and mercury chalcogenide CQDs, as the mainstream materials of CQDs, have excellent infrared detection performance and have become the most ideal materials for infrared photodetectors [22,23,24,25]. Zhao et al. [26] and Hao et al. [27] summarized the recent development of infrared photodetectors based on lead chalcogenide CQDs and mercury chalcogenide CQDs, respectively.

In addition to showing superior performance in the detector field, CQDs also have obvious advantages in spectral filtering [28,29,30]. This indicates that CQDs have great potential for applications in microspectrometers. Qiu et al. [31] presented the advances of micro spectrometers based on material nanoarchitectonics, which pointed to the direction for researchers to study novel low-dimensional materials in this field. Qiao et al. [32] reported that CQDs can be introduced as a sacrificial layer when polishing single-crystal silicon carbide (SiC) using pulsed ion beam sputtering to improve surface quality. This provides a new idea for achieving high-precision fabrication of ultra-smooth single-crystal SiC surfaces.

Low-dimensional materials have a wide range of applications in various fields, such as photovoltaic devices, of which well-designed heterojunctions can make it possible to improve the performance of the devices [33]. In the study by Wang et al. [34], CH_3_NH_3_PbI_3_/Au/Mg_0.2_Zn_0.8_O heterojunction self-powered photodetectors with a high responsivity of 0.58 A/W were demonstrated, where CH_3_NH_3_PbI_3_ and Mg_0.2_Zn_0.8_O acted as the p-type and n-type layer, respectively. This work provides new concepts for the study of perovskite photodetectors with low dark current and high detectivity. Sun et al. [35] reported flexible CZTSSe/ZnO solar cells by optimizing ZnO buffer layers, achieving the maximum power conversion efficiency of 5.0%.

To improve the detection performance of photodetectors, optical structures and photosensitive materials need to be efficiently combined to enhance light absorption. In photoelectric devices, a metal microstructure can be engineered to squeeze light into the sub-diffractive region, and then, the plasmon exciton resonance phenomenon boosts light absorption [36]. Lin et al. [37] investigated a performance-enhanced GaAs nanowire photodetector by introducing Au nanoparticles prepared by thermal evaporation. Assisted by the coupling of electron gas in the Au nanoparticles to the excitation light, the photocurrent and responsivity of this proposed photodetector were raised. Pierini et al. [38] designed a phototransistor that combined two resonances by utilizing a lithium-ion glass gating of HgTe nanocrystal film, realizing a high responsivity.

In addition, the performance and reliability of optoelectronics can also be improved by studying and optimizing the optical material characteristics. Zhao et al. [39] enhanced the homogeneity of large-scale nanorods by controlling the solution flow and tuning the electric field distribution, thus improving their light utilization efficiency. The optimized ZnO NRs have promising applications in solar cells and collector systems. Bai et al. [40] investigated the spatial shifts of the reflected light beam on hexagonal boron nitride (hBN)/alpha-molybdenum (α-MoO_3_) trioxide structure. They successfully enhanced hBN in-plane anisotropy by twisting. This study provides theoretical guidance for novel nanophotonic devices and optical encoders.
